# Lessons from radiation epidemiology

**DOI:** 10.4178/epih.e2018057

**Published:** 2018-11-14

**Authors:** Won Jin Lee

**Affiliations:** Department of Preventive Medicine, Korea University College of Medicine, Seoul, Korea

**Keywords:** Environmental health, Epidemiology, Occupational medicine, Radiation exposure, Risk assessment

## Abstract

Radiation epidemiology has developed as a specialized field and has unique characteristics compared to the other fields of epidemiology. Radiation exposure assessment is highly quantified and health risk assessment can yield precise risks per unit dose in each organ. At the same time, radiation epidemiology also emphasizes the uncertainty of the estimated doses and risks. More radiation epidemiologists work in radiation societies rather than those of epidemiology. This specialization deepens the research of radiation studies but also results in fragmentation from general epidemiology. In addition to continued involvement with radiation-related sciences, therefore, more efforts to communicate with the other fields of epidemiology are necessary for radiation epidemiology.

## INTRODUCTION

Epidemiology has several subdivisions by disease, exposure agent, or research method. Such specialization does not only occur in the field of epidemiology but also in all fields with academic advancements. While this specialization plays a major role in conducting in-depth research in each academic field, there is a limitation of forming fragmentations of the discipline with a reduction in communication with other epidemiologists [1]. Therefore, it is important to understand the advantages and disadvantages of specialization in the field of epidemiology and to explore the development of epidemiology as a whole and its subspecialties. This editorial describes the characteristics of radiation epidemiology to share the areas in which radiation epidemiology is more advanced compared to other areas of epidemiology, while providing an opportunity to overcome the limitations of specialization.

## STATUS OF RADIATION EPIDEMIOLOGY

Radiation epidemiology is a field of epidemiology that deals with the health effects of radiation exposure. Therefore, it is basically an area of environmental and occupational epidemiology. The health effects of radiation have long been reported; however, radiation epidemiology advanced in earnest with the cohort study on the survivors of the 1945 atomic bombing in Japan. The specialized area of radiation epidemiology can further be divided into several areas according to the type of radiation exposure (for example, occupational exposure among radiation workers, medical exposure of the general public and patients, exposure due to disasters or accidents, and exposure to natural radiation). Many studies in each of these specific areas have been conducted and greatly contributed in identifying the health effects of radiation and its management in our society. Despite some uncertainties, major results from radiation epidemiology studies have been summarized as follows: (1) a single exposure can increase cancer risk for life; (2) many small exposures can increase cancer risk; (3) the young are more susceptible than the old; (4) the fetus is not more susceptible than the child; (5) females are more susceptible than males; and (6) risks differ by organ or tissue [2].

Although the term radiation epidemiology is a combination of ‘radiation’ and ‘epidemiology’, they are not of equal importance. Radiation epidemiology is at its root an epidemiologic science, and can hardly be considered a traditional radiation science. However, a gradual increase in the role of radiation itself compared with epidemiology in the process of specialization is observed, leading to a phenomenon similar to judging a person based on the color or shape of his/her coat. This is also a common problem observed in other fields. For example, in the field of infectious disease epidemiology, public health professionals must be knowledgeable about infectious diseases; however, it is more important for researchers to be knowledgeable about epidemiology itself. Sometimes, students majoring in epidemiology and public health do not want to study radiation epidemiology due to the difficulty in understanding radiation. To eliminate this barrier of entry, it should be emphasized that radiation epidemiology is a field of epidemiology that involves concepts of population thinking and group comparisons. Radiation epidemiology should be a branch of epidemiology rather than one of radiology, and this is desirable for the purposes of education for next generations interested in this field. Since radiation is an environmental factor, it is most natural to work closely with environmental and occupational epidemiology groups.

A phenomenon in which radiation itself is emphasized over epidemiology occurs due to the large influence exerted by radiationrelated sciences. When epidemiological questions are asked in radiation sciences, the epidemiological perspective may be neglected if the answers are sought primarily from the perspective of radiology. In reality, there are cases in which the epidemiology-like answers wanted by the radiation fields cannot be fully provided by epidemiology alone. For example, the health effects of low-dose radiation exposure are topics that can be addressed when combined efforts from biology, physics, communications, and philosophy along with epidemiology are exerted. It may be neither desirable nor possible to answer the questions about this topic based on epidemiology alone. It is important to interpret the ‘epidemiology-like’ questions raised in radiation science from an ‘epidemiological perspective’ and to provide the best available answer from the viewpoint of public health.

## CHARACTERISTICS OF RADIATION EPIDEMIOLOGY

### Exposure assessment

Radiation, which is the factor of research in radiation epidemiology, has unique characteristics that differentiate it from other environmental factors. First, our life on earth cannot escape radiation so much as to be able to say that everyone lives in the sea of radiations in terms of exposure (even if one goes into space, one is exposed to more radiation than on earth). Every human being is exposed to the natural radiation emitted by the sun or the earth’s crust every minute of the day. We are also exposed to radiation by medical procedures and from radiation-related work. Although radiation cannot be felt, it is clearly an environmental factor that affects our lives more than air. In this regard, it is impossible to identify non-exposed groups in radiation epidemiological studies. Low-exposure groups could be used for comparison, and radiation exposures other than specific exposure types should be assumed the same or further investigated additonally.

In radiation epidemiology, the exposure assessment is highly quantified; this is the most distinctive characteristic of radiation epidemiology compared with other epidemiological areas. In general epidemiology, the individual measurement data could be considered as accurate exposure indicators. However, in radiation-related disciplines, it is the norm to measure not only individual data but also the amount of radiation entering the body and even the amount entering each organ. These exposure levels per organ become the basic data used for the assessment of organ-specific health effects. Radiation exposure indicators were established for the subdivision into units of effective dose, equivalent dose, and organ dose [3]. Furthermore, lifetime cumulative doses for each individual can be evaluated via DNA analyses. This precise approach to exposure assessment was possible through the contribution of radiation-related sciences, particularly health physics and biology. To the best of my knowledge, no field of epidemiology has ever had such a detailed quantified absolute value for each risk factor. In radiation epidemiology, detailed exposure values can yield absolute values for health risk per dose of exposure, which is a process that is difficult to attempt in other fields of epidemiology. That is, it could have been possible to seek direct answers to not only whether the factor is hazardous but also how much the risk is. This is the huge advantage of radiation epidemiology over other epidemiological areas and it is in line with ideal recommendations of occupational and environmental research [4].

However, it is difficult to obtain a perfect value no matter how accurately the doses are evaluated. Uncertainty exists at each stage of the radiation assessment; therefore, the calculated value only approaches the true value rather than being perfect. Nevertheless, radiation epidemiology is often extremely strict for studies in which the doses have not been assessed. While the studies that calculate the quantified indices are mainly considered worthwhile, those that use relative exposure indices (for example, job title, work duration or department, etc.) may be undervalued and their results may not be obtained for their full significance in public health sometimes. In some cases, however, important clues about causal relationships can be provided even if positive-response relationships are not assessed, and the most compelling studies are not necessarily the most elegant [5]. Radiation epidemiologists need to also seriously consider whether such detailed dose assessments are always necessary for the aforementioned achievements [2]. Continuous quantitative dose assessments are essential for more sophisticated academic development; however, it would be desirable to do so within acceptable limits of epidemiological studies.

### Health effects assessment

Radiation affects almost all body organs. There is sufficient evidence in humans for the carcinogenicity of radiation (X-rays and gamma rays), which include cancers of the salivary gland, esophagus, stomach, colon, lung, bone, basal cells of the skin, female breast, kidney, urinary bladder, brain and central nervous system, and thyroid, and leukemia (excluding chronic lymphocytic leukemia) [6]. Also, radiation exposure is positively associated with cancers of the rectum, liver, pancreas, ovary, and prostate; and non-Hodgkin’s lymphoma and multiple myeloma. Carcinogenicity is often supported by laboratory results of other environmental factors; however, the determination of carcinogenicity due to radiation exposure is mainly based on epidemiological studies that are direct evidence from populations as well as laboratory studies. There are also reports on the relationship between radiation exposure and non-malignant diseases from cardiovascular, eye, thyroid, nervous, digestive, and respiratory systems. The availability of relatively more epidemiological studies than in other environmental factors is another advantage in radiation epidemiology.

In radiation epidemiology, indices of not only the relative risk but also the excess relative risk and absolute risk as well as lifetime attributable risk are calculated to investigate health effects. These precise indicators are possible from the established risk models for cancer as a whole and specific risk models for each organ. In radiation epidemiology, specific risk per unit dose is applied to the risk models considering the age at the time of exposure, sex, time since exposure, and attained age [7]. These detailed risk values provide important information about the risks of radiation in a population (for example, the risks involved when a child is exposed to a computed tomography [CT] scan, and whether this exceeds the advantages of performing CT scan for diagnosis). These models were refined from several cohort studies including the atomic bomb survivor study in Japan, which enrolled about 120,000 individuals (http://www.rerf.or.jp/). Compared to the Framingham Heart Study, which is a traditionally representative cohort study on epidemiology that included approximately 5,000 individuals since 1948, the research methods in radiation epidemiology are actually more innovative. In terms of data analysis, advanced methods have been developed for various models, and even a separate professional statistical program called Epicure was developed for the analysis of the cohort data from the Japanese atomic bomb survivors [8]. This program is currently useful in general cohort studies as well as radiological studies, particularly in calculating the person-years of large volumes of data. These advantages of radiation epidemiology are worth applying to other epidemiological studies.

Although the quantitative absolute risk assessment is clearly a great academic achievement from a public health standpoint, caution must be taken not to prioritize only the quantification of research results at the peril of overlooking the understanding and qualitative approaches to the population being studied. Traditionally, epidemiology is a field of study that tests hypotheses through thoughtful observations and deep thinking, and quantitative technique is just one of the important approaches. Quantitative techniques used to assess risk in epidemiology have developed actively since the 1970s, and have contributed greatly to hypothesis testing and control of confounders. As epidemiologic research involves group comparisons, quantitative methods are essential; however, the quantification of risk assessments is also an endless process, as is the case with exposure assessments. Because the degree of risk can vary depending on the characteristics of the population, timing of exposure, and type of exposure, one should always be prudent with a simple comparison of absolute risk values. In fact, epidemiology may not be a suitable tool for quantitatively differentiating low risk and no risk. Thus, an evaluation of the epidemiological risk of extremely low doses is subject to the limitation as an observational study, and this is not a limitation of the field of epidemiology itself. Risks to small uncertain absolute values can cause unnecessary arguments and lower the significance of epidemiology. Therefore, research that includes quantified outcomes in combination with qualitative analysis can be considered a comprehensive effort that is more faithful to the nature of epidemiology itself.

Regarding the linear no-threshold model, the controversy in the field of radiation could have resulted from differences in concepts at the individual and population levels. That is, there may be a phenomenon in which health effects are not observed in a particular individual below a certain dose but appears once the dosage exceeds the level. However, it may not be reasonable to establish a single threshold for a population because there are several different individuals with different sensitivities. This is not a case of right versus wrong, but a natural aspect of different viewpoints.

### Research on uncertainty

Paradoxically, many debates and studies about uncertainty have been carried out in radiation epidemiology although it has relatively more resources and quantitative indicators than other epidemiological areas. This relates to the way in which radiation epidemiology has progressed to an overly quantified approach because quantification inevitably entails more uncertainty. Radiation epidemiology divides the factors associated with uncertainty into stages (dose estimation process, epidemiological methods, statistical power, risk transfer, dose-dose rate effects, etc.) and assesses the magnitude and direction of uncertainty in each factor [9]. Efforts at finding and reducing the cause of uncertainty are desirable in academic rigidity, and scientific advances have been obtained from them.

In radiation epidemiology, it is important not only to focus on the studies of uncertainty but also to apply them in practice. The principles of what to do when uncertainty exists are proposed and applied at the public health level. Indeed, the International Commission on Radiological Protection (ICRP) contributed greatly in protecting health from radiation by applying the precautionary principle in the 1960s when uncertainty about the radiation effects of leukemia existed [10]. Despite the fact that current radiation epidemiology has far more data and research results than it did in the past, whether uncertainties are being sufficiently applied to realistic issues should be assessed.

### Independent and separated activity

Each country often treats radiation-related areas as a specialized part with a separate, independent, and specialized agency. Internationally, the United Nations Scientific Committee on the Effects of Atomic Radiation under the United Nations and ICRP, which is a professional organization, has a strong influence through a variety of activities, and several research associations specialize in radiation. This environment is clearly a good opportunity to bring radiation-related contents closer together and enable more specialized research by radiation epidemiologists through an indepth understanding of radiation itself.

**Figure 1.**
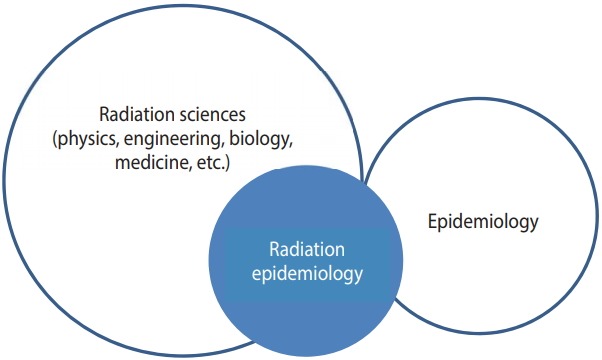
Surrounding environment of radiation epidemiology.

However, being surrounded by more radiation-related professionals than public health professionals has limited the voice of radiation epidemiology (Figure 1). Most radiation epidemiologists have mainly attended the associations of radiation sciences rather than epidemiologic societies. While the use of radiation is intended to be reduced as much as possible in epidemiology, other radiation-related areas often emphasize the use of radiation. The specificity of the radiation field is more likely to monopolize relevant information to specific agencies or companies that use radiation, and naturally, the direction of study from the perspective of using radiation is preferred. In addition, the control of radiation in each country is handled only by specific departments; thus, all projects involving radiation (including epidemiological contents) tend to rely on these specific departments technically and culturally. Therefore, it is important to verify and confirm conflicts of interest in radiation epidemiology, and researchers must be aware of ethical requirements in environmental epidemiology (https://www.iseepi.org/).

These exclusive and authoritative phenomena can appear similarly in the field of research. The current radiation epidemiology considers the results of Japanese atomic bomb survivor studies as the gold standard for interpreting the risk of radiation-induced health effects. This is quite natural in light of the long follow-up periods, detailed exposure assessment and analyses, and abundant research results of these studies. However, excessive reliance on one study may overlook various differences in other studies that are actually being observed and the possibility of other results. Efforts should be exerted to objectively evaluate various radiation epidemiological studies conducted worldwide. Efforts free from the authority and exclusiveness could be further emphasized in the field of epidemiology, which has the central value of open criticism and interpretation.

## CONCLUSION

For the advancement of epidemiology, the specialization of each area is both desirable and inevitable. Radiation epidemiology grew into a specialized division that is closely linked to the field of radiation-related sciences, and these connections are needed for further academic development. At the same time, closer interaction with other epidemiological fields is necessary for maintaining the principles and methods of general epidemiology. It is important to both specialize as a deepening field of study and connect to the general field of epidemiology to apply the basic principles of epidemiology. In terms of temporal prioritization, the early stages of radiation epidemiology primarily needed help from other radiation-related fields; however, at the current stage of radiation epidemiology should be weighted with more epidemiological perspectives. These efforts are helpful not only for radiation epidemiology but also for epidemiology as a whole. From the perspective of general epidemiology, it can be useful in applying the advanced contents of radiation epidemiology, and has the advantage of extending the area of epidemiology by including radiation epidemiology. From the perspective of radiation epidemiology, communication with other fields of epidemiology may be an opportunity to answer radiation-related questions with more in-depth epidemiological concepts and principles.

These issues may apply not only to radiation epidemiology but also to other specialized divisions in epidemiology. It is important for any specialized division of epidemiology to explore ways to foster existing specialization and to strengthen ties with other epidemiological fields. As radiation helps in diagnosing and treating diseases, but also causes diseases, specialization in epidemiology has a variety of advantages and disadvantages. As it is important to understand and use radiation wisely, it is important to develop specialization wisely in epidemiology.
