# Supplementary material for: Lessons from radiation epidemiology
Source: Epidemiol Health. 2018 Nov 14;40:e2018057. doi: 10.4178/epih.e2018057 (PMC6288658; doi:10.4178/epih.e2018057)
Supplement: Supplementary file 1 [file epih-40-e2018057-supplementary.pdf]

Volume: 40, Article ID: e2018057

https://doi.org/10.4178/epih.e2018057

방사선 역학으로부터의 교훈

고려대학교 의과대학 예방의학교실 이 원 진

서론

역학에는 많은 세부 전공분야가 있다. 이러한 현상은 역학분야뿐 아니라 학문의 발전에 따라 모든 분야에서 자연스럽게 나타나고 있다. 사실 일반 역학이라는 것은 추상적인 개념이며 어떤 형태로 던지 (질병종류, 노출형태, 혹은 연구방법 별 등) 전문화된 영역을 통해 '역학 연구'를 구현한다. 이러한 전문화는 각 학문의 연구 내용을 깊게 발전시키는데 큰 역할을 하고 있는 반면, 일반 역학 분야와의 교류가 부족해지면서 연구 영역이 분열되는 단점도 있다 [1]. 따라서 역학분야에서 전문화 현상의 장단점을 파악하고 이를 근거로 전체 역학 및 각 전문 분과의 발전을 모색하는 것이 중요하다. 이 글에서는 역학의 전문 분과의 하나인 방사선 역학을 사례로 그 장단점 및 특성을 정리함으로써 다른 역학 분야에 비해서 발전된 부분을 공유하여 다른 한편으로는 전문화에 따른 단점을 극복할 수 있는 계기를 마련하고자 한다.

방사선 역학의 현재

방사선 역학은 방사선이라는 환경 인자의 분포 및 노출로 인한 건강영향을 다루는 역학의 한 분야이다. 따라서 기본적으로 환경 및 직업역학의 한 분야라고 할 수 있다. 방사선에 의한 건강 영향은 오래 전부터 보고되어 왔으나 방사선 역학은 1896년 엑스선 발견 이후 다른 방사선 관련 학문들 (물리학, 공학, 생물학, 의학 등)의 활발한 연구결과와 함께 시작되었고, 1945년 일본 원폭을 계기로 시작된 원폭생존자 코호트 연구를 계기로 본격적으로 발전하였다고 할 수 있다. 방사선 역학이라는 전문분야 안에서도 방사선 노출 유형에 따라 여러 영역으로 나뉘어진다 (예, 방사선 종사자들의 직업적 노출에 대한 분야, 일반인 및 환자들의 의료 노출 분야, 재해나 사고로 인한 노출 분야, 자연 방사선 노출 연구 분야 등). 이러한 각 세부 분야별로 많은 연구들이 진행되었고 이를 통해 방사선에 의한 건강영향을 밝히고 우리사회에서 방사선을 관리하는데 큰 기여를 하여 왔다. 지금까지 방사선 역학 연구들을 통해 얻은 주요 결과들은 일부 불확

Volume: 40, Article ID: e2018057

https://doi.org/10.4178/epih.e2018057

실성이 있지만 1) 방사선에 단 한번의 노출도 평생 암 위험도를 증가시킨다 2) 적은량의 여러번 방사선 노출은 암 위험도를 증가시킨다 3) 나이가 어릴 때 노출될수록 방사선의 위험도가 크다 4) 태아 노출은 소아 노출보다 위험도가 크지 않다 5) 여성이 남성보다 민감하다 6) 방사선에 의한 각 장기의 위험도가 다르다 등으로 개략 요약된 바 있다 [2].

방사선 역학은 '방사선'과 '역학'이라는 두 단어의 조합이지만 그 중요성은 두 영역이 동등하지 않다. 즉 방사선 역학은 기본적으로 역학이며 전통적인 방사선 과학이라고 보긴 어렵다. 그러나 전문화 과정에서 역학보다는 방사선 자체의 비중이 커지는 현상이 점차 발생되면서 마치 외투의 모양이나 색깔을 보고 그 안의 사람을 간과하는 현상이 나타난다. 이러한 모습은 다른 전공에서도 비슷하게 나타나는 공통적인 문제점이기도 하다. 예를 들어 전염병역학에서도 공중보건학 전공자가 전염병을 아는 것도 중요하지만 역학 자체를 아는 것이 더 중요하다. 간혹 역학 및 보건학을 전공하는 학생들이 방사선을 이해해야 하는 어려움 때문에 방사선 역학에 깊이 들어오지 못하는 모순된 경우도 있다. 이러한 진입장벽을 없애기 위해서라도 방사선 역학은 기본적으로 집단적 사고와 집단 비교라는 개념을 견지하고 있는 역학이라는 것을 강조해야 한다. 방사선 역학은 방사선학보다는 역학의 한 분과가 되어야 하며 이것은 역학에 관심을 가진 후속세대들의 교육적 목적을 위해서도 바람직하다. 방사선이 환경인자 중 하나이므로 전체 역학분야에서도 특히 환경 및 직업 역학 관련 학술단체들과 긴밀히 함께 활동하는 것이 가장 자연스럽다고 할 수 있다.

방사선 자체가 역학보다 먼저 강조되는 현상은 역학이 방사선 관련 학문들에 둘러싸여 있기 때문에 발생된다고 할 수 있다. 방사선 관련 학문들 속에서 역학적 질문들이 던져졌을 때 이것을 방사선학적 관점에서 우선적으로 답을 찾으려다 보면 자칫 역학적 입장이 소홀해 질 수 있다. 사실 일반적인 방사선 학문들에서 원하는 역학스러운 대답은 역학만으로 완전한 답을 줄 수 없는 경우들이 있다. 예를 들어 저선량 방사선 노출에 의한 건강영향은 역학을 포함한 생물학, 물리학, 심지어 의사소통 및 철학적 분야까지 함께 노력해야지 해결될 수 있는 질문이다. 이것을 역학 혼자 짊어지려는 것은 바람직하지도 가능하지도 않다. 방사선 과학에서 제기되는 '역학스러운' 질문들을 '역학적' 입장에서 해석하고 최선의 답을 제시되는 것이 중요하다.

방사선 역학의 특성

1) 노출 및 평가의 특이성

방사선 역학에서의 연구대상 인자인 방사선은 다른 환경 인자와 구별되는 특성이 있다. 우선 노출 면에서 모든 사람들이 방사선의 바다 속에 살고 있다고 할 정도로 지구상의 우리 삶은 방사선을 벗어날 수 없다 (설령 우주로 나간다 하더라도 지구에서보다 훨씬 더 많은 방사선에 노출된다). 즉 모든 인간은 일상생활에서 지각이나 태양에서 방출되는 자연방사선에 매 순간 노출되고 있다. 직업적으로 방사선을 다루는 사람들은 말할 것도 없고 의료기관에서 진단과 치료과정에서도 많은 사람들이 방사선에 노출되고 있다. 방사선은 느낄 수 없지만 공기보다도 더욱 밀접히 우리 삶을 둘러싸는 환경 인자인 것이 분명하다. 이런 면에서 방사선 역학 연구에서 비노출 집단을 찾는 것은 불가능하다. 저노출군을 비교대상으로 하거나 특정 노출 형태 이외의 방사선 노출에 대해서는 추가적으로 조사하거나 동일하다고 가정해야 한다.

방사선 역학에서는 노출에 대해 매우 정량화된 평가 체계를 갖고 있다. 이것은 방사선 역학이 다른 역학 분야에 비교해서 가장 두드러지게 만드는 특성이라고 할 수 있다. 일반 역학에서는 개별 사람들에 대한 측정 자료가 있는 것만으로도 정확한 노출지표라고 간주되고 있는데 반해, 방사선 관련 학문분야에서는 개인 측정자료를 넘어서 신체에 들어온 선량 그것도 각 장기별 선량값까지 산출하는 것이 일반화되어 있다. 이러한 장기별 노출 지표는 각 장기별 건강 영향을 평가할 수 있는 근거자료가 된다. 이에 따라 방사선 노출 지표가 유효선량, 등가선량, 장기 선량 등의 단위로 각기 세분화되어 만들어졌다 [3]. 더 나아가서 개인에 대한 평생 누적 선량이라는 지표를 혈액 분석을 통해 평가하기도 한다. 이러한 노출평가에 대한 정밀한 접근은 방사선 관련 학문, 특히 보건물리학 및 생물학의 기여를 통해 가능하였다. 내가 아는 한 지금까지 어떤 역학 분야도 각 위험인자에 대해 이렇게까지 자세한 정량화된 절대값을 갖진 못하였다. 방사선역학에서는 자세한 노출값을 통해 다른 역학에서는 시도하기 어려운 선량 노출당 건강위험도에 대한 절대값을 산출할 수 있다. 즉 해당 인자의 위험성 여부를 넘어서 얼마나 위험한지에 대한 직접적인 답을 추구하는 것이 가능할 수 있었으며, 이것은 다른 역학분야에서 보다 방사선 역학이 갖는 매우 큰 장점이며 권장되고 있는 바람직한 직업 및 환경 역학연구의 방향과 일치한다 [4].

그러나 아무리 정교하게 선량을 평가한다고 하더라도 절대적으로 완벽한 값을 얻기는 어렵다. 선평 평가의 각 단계마다 불확실성이 존재하여 산출된 값이 완벽하기 보다는 참값에 근접해 들어가는 것일 뿐이다. 그럼에도 불구하고 방사선 역학은 선량이 평가되지 못한 연구들에 대

https://doi.org/10.4178/epih.e2018057

해서 지나칠 정도로 엄격한 경우가 있다. 즉 장기 선량 등의 정량적 지표를 산출한 연구들만을 강조하여 일반적인 다른 역학분야에서와 같이 상대적 노출 지표들 (예, 직업력, 작업부서, 기간 등)을 사용한 연구들에 대해 과소평가함으로써, 그 결과들에 대한 보건학적 의미를 충분히 살리지 못하는 측면이 있다. 경우에 따라 양-반응 관련성을 평가하지 못한다고 하더라도 인과관계에 중요한 단서를 제공할 수도 있으며, 가장 중요한 연구들이 가장 정교할 필요는 없다 [5]. 방사선 역학자들은 앞서 언급된 그 동안의 성과들을 [2] 위해서 과연 그토록 자세한 선량 평가가 반드시 필요했던가 라는 것을 진지하게 생각해 볼 필요도 있다. 지속적인 정량적 선량 평가는 보다 정교한 학문적 발전을 위해 반드시 필요하지만 역학 연구의 허용가능한 범위 내에서 합리적으로 이루어지는 것이 바람직하다.

2) 건강영향 평가의 정교함

방사선은 거의 전 신체기관에 건강영향을 초래한다. 악성 종양과 방사선 (엑스선 및 감마선)과의 관련성에 있어서는 침샘암, 식도암, 위암, 대장암, 폐암, 골암, 피부기저세포암, 여성유방암, 신장암, 방광암, 뇌종양 및 중추신경계암, 갑상선암, 백혈병 (만성림프구성 백혈병 제외) 등이 인체발암성에 충분한 근거 (sufficient evidence)가 있는 것으로 평가되었으며, 직장암, 간암, 췌장암, 난소암, 전립선암, 비호치킨림프종, 다발성골수암 등은 인체발암성이 양관련성 (positive association)으로 평가되었다 [6]. 방사선 노출에 의한 이러한 발암성 평가는 다른 발암물질들과 달리 실험실적 연구결과들뿐 아니라 직접적인 인구집단을 대상으로 한 많은 역학연구 결과들에 근거하고 있다. 그 외에도 심혈관계, 백내장, 갑상선, 신경계, 소화기, 호흡기계 등 비발암성 질환과의 관련성에 대한 많은 보고들이 있다. 방사선 관련 건강영향 결과들은 다른 환경인자들보다 상대적으로 많이 진행된 인구 집단에 대한 역학 연구 결과들에 의해 뒷받침되고 있다는 장점이 있다.

방사선 역학에서는 상대위험도뿐 아니라 초과상대위험도 및 초과절대위험도, 그리고 발암모델에 기반하여 생애추가위험도라는 지표를 산출함으로써 보다 정밀하게 건강 영향에 대한 분석을 실시하고 있다. 이러한 지표들의 산출은 이미 많은 역학 연구들을 통해 정교한 모델들이 만들어졌고 다듬어지고 있기 때문에 가능하다. 즉 암에 있어서는 전체 고형암에 대한 위험도 모델뿐 아니라 각 장기별 특이적인 위험도 모델도 만들어져 있으며, 이러한 모델들에는 노출시 연령, 성별, 노출 이후 시간 및 도달 연령 등을 고려한 특이적인 위험계수를 적용되고

있다 [7]. 이처럼 정교한 위험도 값은 인구집단에서 방사선의 위험도에 대한 자세하고 중요한 정보를 제공해준다 (예를 들어 어린이가 CT에 노출되었을 때 과연 얼마나 위험도가 있는지 그리고 그것이 진단을 위해 촬영되는 CT의 이득을 초과하는지 등). 이러한 정교한 모델이 가능했던 것은 일본 원폭을 계기로 만들어진 약 12 만명에 대한 대규모 전향적 코호트 연구를 비롯하여 (http://www.rerf.or.jp/) 다수의 방사선 노출자 코호트 연구들이 진행되었기 때문이다. 이는 역학에서 고전적으로 대표되는 코호트 연구인 Framingham Heart Study가 약 5,000명을 대상으로 1948년부터 진행된 것과 비교했을 때 뒤지지 않은 오히려 선도적인 연구방법의 형태이다. 자료 분석적인 면에서도 여러 모델 개발과 관련하여 앞선 방법들이 개발되었으며 일본원폭 생존자 코호트 자료 분석을 위해 Epicure라는 별도의 전문적인 통계 프로그램까지 개발된 바 있다 [8]. 이 프로그램은 현재 방사선 역학에서 만이 아니라 일반적인 코호트 연구 분석시, 특히 대용량 자료의 인년 (person year) 산출에 유용하게 활용되고 있다. 이러한 방사선 역학의 장점들은 다른 역학연구 분야에 활발히 응용될 충분한 가치가 있다.

정량적인 절대 위험도 평가는 위대한 학문적 성과임에는 분명하지만 공중보건학적 입장에서 연구결과가 지나치게 정량화에만 치중하고 연구대상 인구집단에 대한 이해 및 정성적 접근을 간과하는 것은 주의해야 한다. 역학은 전통적으로 자세한 관찰과 깊이 있는 사고를 통해 가설을 검증하는 학문으로서 정량적인 측면은 중요한 한 부분일 뿐이다. 역학에서 위험도에 대한 정량적인 기법은 1970년대 이후 활발히 발전된 분야로서 가설검정 및 교란 변수 통제 등에 큰 기여를 하여왔다. 역학이 기본적으로 집단 비교를 하는 연구이므로 정량적 방법은 필수적이지만 위험도 평가의 정량화는 노출평가에서와 마찬가지로 끝이 없는 과정이기도 하다. 그리고 위험도는 각 인구집단의 특성, 노출 시기 및 노출 형태에 따라 차이를 보일 수 있어 단순한 절대 위험도값 비교는 항상 신중해야 한다. 사실 역학은 정량적으로 위험도가 없는 것과 (no risk) 위험도가 적은 (low risk) 것을 구별하는데 적합한 도구는 아닐 수 있다. 따라서 지극히 낮은 저선량에 대한 역학적 위험도 평가는 관찰연구로서의 많은 제한을 가질 수 밖에 없으며 이것이 역학이라는 학문 자체의 제한점은 아니다. 불확실한 작은 절대값에 대한 위험도는 불필요한 논쟁을 야기시키고 오히려 역학이 갖고 있는 중요한 의미를 희석 시킬 수 있다. 따라서 정량화된 결과를 정성적 해석과 함께 포괄하여 연구하는 것이 역학 본연의 특성에 보다 충실한 종합적인 노력이라고 할 수 있다.

Volume: 40, Article ID: e2018057

https://doi.org/10.4178/epih.e2018057

방사선 영역에서 논쟁이 되고 있는 문턱없는 선량모델 (LNT)의 경우도 개별적 차원과 집단적 차원의 개념차이에서 비롯되었다고 할 수 있다. 즉 특정 개인에서 특정 선량 이전에는 건강 영향이 나타나지 않다가 그 이상에서 영향이 나타나는 현상은 존재할 수 있다. 그러나 인구집단에는 민감도가 다른 많은 다양한 개인들이 존재하기 때문에 집단을 대상으로 한 단일한 역치를 설정하는 것은 합리적이지 않을 수 있다. 이러한 두 가지 사실은 무엇이 맞고 틀리는 것이 아니라 관점에 따른 자연스러운 다른 모습일 뿐이다.

3) 불확실성에 대한 활발한 연구

방사선 역학은 다른 역학분야들보다 상대적으로 풍부한 자료원과 정량적 지표들을 갖고 있음에도 불구하고 역설적으로 불확실성에 대한 연구와 논의가 많이 진행되어 왔다. 이것은 방사선 역학이 지나치게 정량화된 접근방법으로 진행해온 것과 관련되어 있다. 정량화로 갈수록 더 많은 불확실성이 필연적으로 수반될 수 밖에 없기 때문이다. 방사선 역학에서는 불확실성에 관련되는 요인들을 각 단계별로 구분하고 (선량 산출과정, 역학연구 방법, 통계적 검정력, 위험도 전이, 선량-선량률 효과 등) 각 요인들에서의 불확실성의 크기와 방향을 평가하고 있다 [9]. 이처럼 불확실성의 원인을 찾고 줄여나가는 노력은 학문적 엄격성에서 바람직하며 이를 통해 많은 과학적 진전을 이루어 왔다.

방사선 역학에서는 불확실성 연구 자체에 집중하는 것뿐 아니라 이것을 실제에 적용하는 것도 중요하다. 불확실성이 존재할 때 어떻게 해야 하는지에 대해서는 이미 몇 가지 원칙들이 보건학적 차원에서 제안되고 적용되고 있다. 실제로 ICRP에서는 1960년대에 백혈병에 대한 방사선 영향에 불확실성이 존재했을 때 사전예방주의 원칙을 적용하여 방사선으로부터 건강 보호에 큰 기여를 한 바 있다 [10]. 현재의 방사선 역학은 과거보다 훨씬 더 많은 자료와 연구결과를 갖고 있음에도 불구하고 불확실성을 현실적 이슈들에 충분히 적용하고 있는지 되돌아 보아야 할 것이다.

4) 독립되고 분열된 활동

각 국가는 방사선 관련분야를 특수한 영역으로 간주하여 독립되고 전문화된 별도의 관련기관을 갖고 있는 경우가 많다. 국제적으로도 유엔산하의 UNSCEAR 및 전문 단체인 ICRP 등이

Volume: 40, Article ID: e2018057

https://doi.org/10.4178/epih.e2018057

다양한 활동을 통해 큰 영향력을 행사하고 있으며, 그 외 여러 방사선 전문 학술단체들이 있다. 이러한 환경은 방사선 관련 내용들을 서로 밀접히 접목시킬 수 있는 좋은 기회임에 틀림없고 방사선 자체에 대한 깊이 있는 이해를 통해 방사선 역학자들이 더욱 전문적인 연구를 가능하게 한다.

현재 방사선 역학은 방사선학 관련 전문분야들이 주도적인 환경에서 (그림 1) 자기 목소리를 내는데 제한적인 상황이다. 현재 대부분의 방사선 역학자들은 역학회보다도 방사선학회의 일부 분과에서 활동하는 경우가 많다. 역학에서는 방사선의 사용을 가능한 줄이려는 입장인 반면 다른 방사선 관련 분야들은 방사선의 사용을 강조하는 경우가 많다. 방사선 분야의 특수성으로 인해 방사선을 사용하는 특정 기관이나 회사에 관련 정보가 독점되는 경향이 있으며, 자연스럽게 방사선을 사용하는 입장에서의 연구방향을 선호하게 된다. 또한 각 국가에서 방사선에 대한 관리가 특정 부서에서만 다루어지고 있어 방사선에 관련된 모든 사업들이 (심지어 역학적 내용까지) 특정 부서에 독점적으로 의존하는 기술적 문화적 경향들이 존재한다. 따라서 방사선 역학에서는 이해충돌을 검증하고 확인하는 것이 중요하며 관련 연구자들은 환경 역학에서의 윤리 규정을 유념할 필요가 있다 (https://www.iseepi.org/).

(Fig 1 here)

이러한 독점적이고 권위적인 현상은 연구 분야에서도 비슷하게 나타날 수 있다. 즉 현재 방사선 역학연구에서 일본원폭생존자 연구 결과를 방사선에 의한 건강 영향의 위험도를 해석하는데 교과서적인 기준으로 삼고 있다. 이것은 일본원폭생존자연구의 오랜 추적 기간, 정교한 노출평가와 분석 및 많은 연구결과에 비추어 볼 때 매우 당연하다. 그러나 한 연구에 지나친 의존은 실제 관측되고 있는 다른 연구들의 다양한 차이 그리고 다른 결과의 발생 가능성을 자칫 간과할 수 있다. 일본원폭생존자 연구를 보다 입체적으로 바라보기 위해서라도 전 세계에서 진행되는 다양한 방사선 역학연구들을 객관적으로 비교 평가하려는 노력이 필요하다. 독점과 권위로부터 탈피하려는 노력과 태도는 자유로운 비판과 해석을 핵심 개념으로 하고 있는 역학이라는 학문에서 더욱 강조된다.

결론

역학의 발전을 위해서 각 분과가 보다 전문화되는 것은 바람직하며 필연적이다. 방사선 역학에서도 방사선 관련 학문 분야와 더욱 밀접히 연계하여 깊이 있는 전문 분과로 성장할 수 있었으며 앞으로도 이러한 연계는 학문적 발전을 위해 필요하다. 이와 동시에 일반 역학의 원칙과 방법을 지켜나가기 위해서 기존 역학 분야와 보다 긴밀한 교류가 필요하다. 즉 세부 학문 분야로서 깊이 있게 전문화되는 것과 동시에 전체 역학 분야와의 연계를 통해 역학의 기본 원칙을 발전시키는 것이 모두 중요하다. 그러나 시간적 우선 순위를 생각한다면 학문 발전의 초기 단계에서는 다른 방사선 관련 분야의 도움이 우선적으로 필요 했었다면, 현재는 그러한 결과들을 역학적 관점에서 발전시켜 나가려는 노력이 더 필요한 시점이라고 할 수 있다. 이러한 노력은 방사선 역학뿐 아니라 전체 역학의 발전 방향에도 부합된다. 우선 다른 분야의 역학 분과 입장에서는 방사선 역학의 발전적인 내용을 받아들여 활용하는 것은 유용한 도움이 될 수 있다. 또한 방사선 역학 분야를 포괄함으로써 역학 전반의 외연을 넓히는 장점이 있다. 한편 방사선 역학 입장에서는 일반 역학과의 교류를 통해 방사선 관련 질문들에 대해 보다 역학적 개념과 원칙에서 깊이 있는 연구들 진행할 수 있는 계기가 될 수 있다.

이러한 제안은 단지 방사선 역학만이 아니라 역학의 각 전문 분과들에 모두 적용될 수 있다. 전문화된 다양한 역학의 각 분과들은 기존의 전문성을 더욱 살려 나가는 동시에 다른 역학 분야와의 연계를 강화시키는 방법을 서로 모색하는 것이 중요하다. 방사선이 질병을 진단하고 치료하는데 도움이 되고 있음과 동시에 질병을 유발하고 있듯이 역학의 전문화는 여러 장단점을 동시에 갖는 다양한 얼굴을 하고 있다. 방사선을 올바로 이해하고 잘 활용하는 것이 중요하듯 전문화 현상을 잘 살리는 것이 중요하다.

참고문헌

1. Bhopal R. Concepts of epidemiology. 3rd ed. London: Oxford University Press; 2016, p. 402-403.

2. Boice JD Jr, Lauriston S. Taylor lecture: radiation epidemiology--the golden age and future challenges. Health Phys 2011;100:59-76.

3. International Commission on Radiological Protection (ICRP). ICRP publication 103:

https://doi.org/10.4178/epih.e2018057

the 2007 recommendations of theinternational commission on radiological protection; 2007 [cited 2018 Oct 20]. Available from: http://www.icrp.org/publication.asp?id=ICRP%20Publication%20103.

4. Loomis D. On the importance of quantification. Occup Environ Med 2012;69:609.

5. Wilcox AJ, Savitz DA, Samet JM. A tale of two toxicants: lessons from Minamata and Liaoning. Epidemiology 2008;19:1-2.

6. International Agency for Research on Cancer (IARC). IARC monographs on the evaluation of carcinogenic risks to humans: volume 100D [cited 2018 Oct 20]. Available from: https://monographs.iarc.fr/iarc-monographs-on-the-evaluation-of-carcinogenic-risks-to-humans-18/.

7. National Research Council. Health risks from exposure to low levels of ionizing radiation: BEIR VII phase 2; 2006 [cited 2018 Oct 20]. Available from: http://www.philrutherford.com/Radiation_Risk/BEIR/BEIR_VII.pdf.

8. Preston DL, Lubin JH, Pierce DA, McConney M, Shilnikova N. Epicure risk regression and person-year computation software: command summary and user guide. Ottawa: Risk Sciences International; .2015.

9. National Council on Radiation Protection and Measurements. Report No. 171. Uncertainties in the estimation of radiation risks and probability of disease causation; 2012 [cited 2018 Oct 20]. Available from: https://ncrponline.org/shop/reports/report-no-171-uncertainties-in-the-

https://doi.org/10.4178/epih.e2018057

estimation-of-radiation-risks-and-probability-of-disease-causation/.

10. International Commission on Radiological Protection (ICRP). ICRP publication 9: recommendations of the ICRP; 1966 [cited 2018 Oct 20]. Available from: http://www.icrp.org/publication.asp?id=ICRP%20Publication%209.

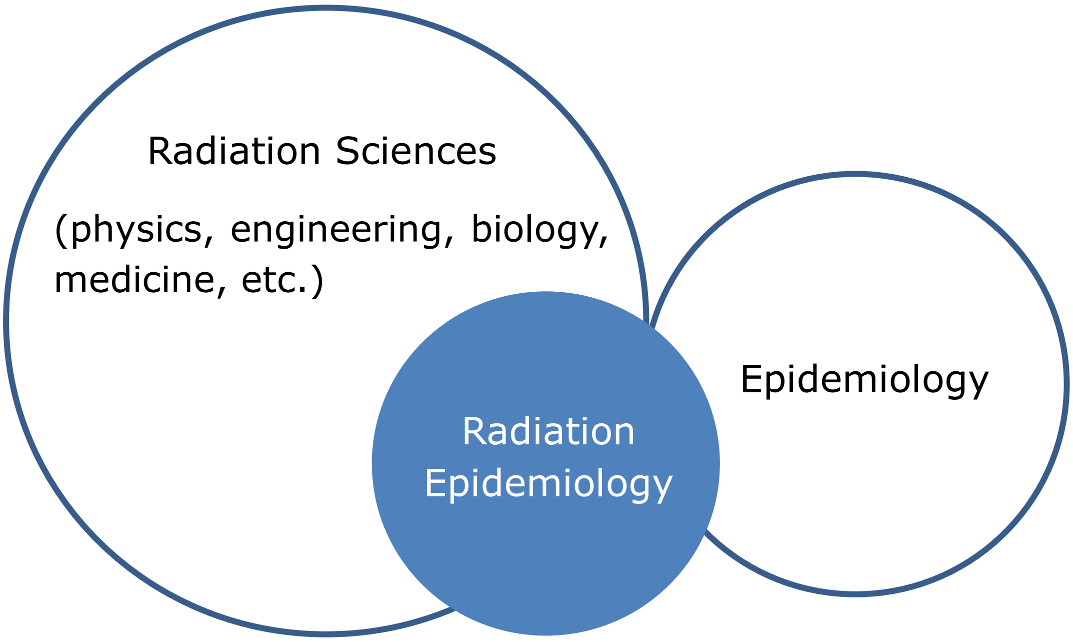

Figure 1. Surrounding environment of radiation epidemiology.
